# Physiological Impacts of Cold Conditions during Moderate Intensity Activity while Wearing Firefighter Protective Clothing

**DOI:** 10.1017/S1049023X25101507

**Published:** 2025-10

**Authors:** Andrew R. Poreda, Kyle J. Kelleran, Caitlin M. Delaney, Cameron P. DeMott, Nan Nan, Chang-Xing Ma, Brian M. Clemency, David Hostler

**Affiliations:** 1. Department of Emergency Medicine, Jacobs School of Medicine and Biomedical Sciences, University at Buffalo, Buffalo, New York USA; 2. Department of Biostatistics, School of Public Health and Health Professions, University at Buffalo, Buffalo, New York USA; 3. Department of Exercise and Nutrition Sciences, School of Public Health and Health Professions, University at Buffalo, Buffalo, New York USA

**Keywords:** Emergency Medical Services, firefighting, frostbite, hyperthermia, hypothermia

## Abstract

**Introduction:**

Thermal protective clothing (TPC) protects firefighters from physical threats associated with structural firefighting. However, it also limits the release of body heat generated, which can result in hyperthermia and dehydration. Despite the prevalence of winter structure fires in the United States, there is a paucity of cold-weather firefighting research.

**Study Objective:**

This study documented physiological responses to moderate-intensity exercise in a cold environment while wearing TPC with the hypothesis that while exercising in firefighting TPC, a cold environment would maintain normal core body temperature and decrease extremity temperature compared to a thermal neutral environment.

**Methods:**

Fourteen firefighters (two females; 30.9 [SD = 8.1] years) participated in both a thermal neutral (20°C) and cold (-8°C) condition simulation. Each subject was outfitted with a heart rate (HR) monitor, eight surface temperature sensors, and a core temperature (Tc) capsule prior to donning TPC. For each condition, subjects walked on a treadmill in an environmental chamber to simulate the common firefighting work intervals of two 20-minute sessions, with a short rest in between, followed by a 20-minute rehabilitation period. Body temperatures, HR, respiratory rate (RR), rate of perceived exertion (RPE), and thermal sensation, comfort, and preference were recorded during exercise and recovery.

**Results:**

Core temperature, HR, RR, and RPE increased during exercise in both conditions. Mean skin temperature (MST) rose during the thermal neutral condition but not during the cold condition. Overall, Tc (0.3 [SD = 0.4]°C; P = .0142), HR (26.3 [SD = 8.36] BPM), RR (3.56 [SD = 5.6] BPM), RPE (2.0 [SD = 1.9]), and MST (3.4 [SD = 1.2]°C) were all higher at the end of the neutral condition compared to the cold condition. During recovery, most measures returned to baseline after approximately five-to-20 minutes in both conditions, but they recovered more slowly in the thermal neutral condition.

**Conclusion:**

Moderate-intensity exercise in TPC increased physiological and perceptual measures more in a thermal neutral environment than a cold environment. Recovery was faster following the cold condition. This may allow firefighters to work for longer durations or recover faster, possibly allowing for fewer crews on scene. However, this study did not account for the risk of other cold induced conditions due to prolonged exposure, such as frostbite. Further investigations should be conducted on cold weather firefighting and its impact on firefighters to establish guidelines and standard operating procedures.

## Introduction

The physiological demands of fire suppression activities in thermal neutral and hot conditions are well-documented.^1,2^ Little is known, however, about the magnitude of the physiological response to fire operations in cold conditions.^1^ Additionally, there are few peer-reviewed reports of post-exertion rehabilitation following cold weather firefighting operations.^1^

Firefighter thermal protective clothing (TPC) is comprised of three layers: the outer shell, which protects against abrasions and cuts, a moisture barrier, and a thermal barrier.^3^ These layers protect the firefighter from heat and physical trauma associated with structural firefighting. However, TPC also limits the release of body heat generated during strenuous physical activity, which can overwhelm the thermoregulatory system resulting in hyperthermia and hypohydration.^1,2,4^ The mass of TPC and self-contained breathing apparatus (SCBA), and the extra effort associated with breathing through a SCBA regulator, will increase workload and physiological responses including heart rate (HR), respiratory rate (RR), and oxygen consumption.^5-7^ Each of these factors – heat retention, worn mass, and restricted breathing – all reduces firefighter performance in thermal neutral and hot environments.^1,2,4-7^ Moisture and thermal barriers incorporated into TPC trap sweat and restrict evaporative cooling, resulting in a microclimate that can reach 118°F and 100% humidity, potentially leading to progressive hyperthermia.^8^ Rising core temperature (Tc) activates the sweat response causing the firefighter to lose body mass and plasma volume.^1,2^

Fires are more likely to result in fatalities during winter months.^9^ In the United States annually, there are over 150,000 fires resulting in an estimated 2,600 injuries and 630 deaths during the winter months.^10^ Despite this, the physiological responses to exertion in cold weather while wearing TPC has not been evaluated. National Fire Protection Association (NFPA; Quincy, Massachusetts, USA) 1971 serves as the standard for the design and testing of firefighting protective gear, but cold weather protection is not addressed for TPC beyond a small statement dedicated to gloves.^3^ Because there are insufficient data regarding physiological responses to cold weather firefighting operations in TPC, best practices are often extrapolated from other fields.^1^ Rissanen, et al examined similar dilemmas in Finnish soldiers wearing nuclear, biologic, and chemical (NBC) protective gear while performing activities in the cold outdoor air.^11^ The NBC protective gear is known to cause heat retention like firefighter TPC.^12^ Rissanen, et al reported that Tc remained stable or elevated based on the degree of exertion.^11^ However, the temperature of the fingers were dependent on the ambient air temperature, and instead of rising in tandem with the rising metabolic rate of exertion, dropped with lowering outdoor temperatures.^11^

To the authors’ knowledge, there are no studies addressing the impact of TPC use in cold weather. The current study measured the heat strain and physiological responses during moderate-intensity exercise in TPC. It was hypothesized that, while exercising in firefighting TPC, a cold environment would maintain normal core body temperature and that extremity temperature would decrease, due to the physiological peripheral vasoconstriction and blood shunting to the core, compared to a thermal neutral environment.

## Methods

This was a laboratory-based, crossover design study of the thermal and cardiovascular effects of cold conditions on firefighters while wearing TPC. The protocol mimicked common intervals of fire suppression activity in which a firefighter works two 20-minute intervals, separated by a short rest period, followed by a longer recovery interval (eg, emergency incident rehabilitation). This study was approved by the University at Buffalo Institutional Review Board (Buffalo, New York USA), ID#: STUDY00006323.

Subjects were recruited from the Western New York region via standard recruitment flyers and social media posts, and were eligible for inclusion if they were aged 18 to 45 years, held active interior firefighter qualifications, and had current medical screening through their respective agencies as part of that qualification. Exclusion criteria included pregnancy, cardiac or respiratory disease, medications known to alter cardiac response to exercise or thermoregulation, musculoskeletal disorders or current injury, renal disease, Reynaud’s phenomenon, diabetes, hypertension, previous cold injury, or other circulatory disease. Subjects were also excluded if body weight was less than 80lbs, or a contraindication to the use of the CorTemp Sensor (HQ, Inc.; Palmetto, Florida USA). Twenty firefighters (three female) from career and volunteer fire departments provided informed consent and began at least one experimental visit. All subjects underwent additional medical screening, including a health history questionnaire and a study-specific medical questionnaire. Anthropometric data (height, mass, and body-mass index [BMI]) and resting vital signs including HR, RR, and blood pressure were also collected.

### Experimental Protocol

The study included two protocol visits, one for each condition (thermal neutral and cold). Participants were assigned trial conditions in a counter-balanced order. Each experimental condition was conducted in a climate-controlled 4.0x2.4m environmental chamber and separated by at least 48 hours. Prior testing of the environmental chamber had suggested that the lowest reliable temperature setting was -8.0°C (17.6°F), which would be a temperature that is commonly found during a United States winter.

Subjects were instructed to abstain from alcohol, nicotine, and caffeine for at least 12 hours prior to testing. Euhydration was verified by urine specific gravity (USG) (Master Hand-Held Refractometer, Atago Co.; Tokyo, Japan). The visit was rescheduled if USG was not less than 1.025. Female subjects completed a pregnancy test at the start of each visit. Subjects were weighed on a digital balance (Minebea Intec, Midrics 2; Holbrook, New York USA) and had their height measured on a standard stadiometer wearing a t-shirt, socks, and athletic shorts. To ensure adequate and standardized pre-exercise nutrition, each subject was given 1g/kg of a meal replacement bar (CLIF Bar; Berkley, California USA) and 500mL of water approximately one hour prior to exercise.

**Figure 1.**
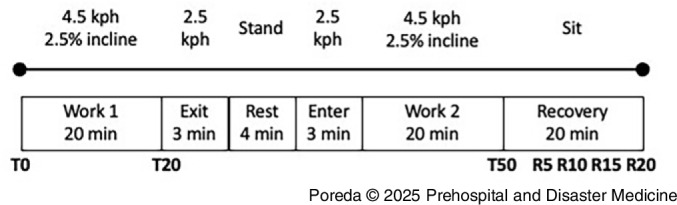
Representation of Experimental Protocol. Note: Subjects walk on a treadmill for two 20-minute intervals, separated by a slower walk away from operations, a standing rest, then a return to the incident. The second work interval was followed by a seated 20-minute recovery period with TPC removed. T = measurement point during exertion. R = measurement point during recovery. Abbreviation: TPC, thermal protective clothing.

Subjects were instructed to ingest a capsule eight hours prior to arrival in the lab to measure gastrointestinal temperature.^13,14^ Upon arrival, subjects were also outfitted with an HR monitor (Polar Electro Oy; Kempele, Finland) around their torso and eight skin temperature sensors (iButton, Maxim Integrated; San Jose, California USA) over the right pectoralis major (chest), the right infraspinatus muscle (back), the midpoint of the right triceps brachii (arm), and the midpoint of the right quadriceps femoris (thigh) for mean skin temperature (MST) calculation.^15^ Skin temperature sensors were also placed on the dorsum of each hand over the midpoint of the third metacarpal (hand) and on the ventral side of the third distal phalanx (finger) of each hand.^16^ The sensors were secured to the skin with medical tape. The subjects donned a standardized set of TPC consisting of turnout pants, coat, and polycarbonate helmet (Lion Group, Inc.; Dayton, Ohio USA), a Nomex hood (Majestic Fire Apparel, Inc.; Lehighton, Pennsylvania USA), rubber boots (Thorogood USA; Merrill, Wisconsin USA), and leather gloves (Shelby Glove; Glenwood, Arkansas USA). The subjects wore a SCBA, including a cylinder and mask (MSA Safety, Inc.; Cranberry Township, Pennsylvania USA), but were not connected to an air supply. Subjects breathed ambient air throughout the protocol (Figure 1).

Rate of perceived exertion (RPE) was measured with the OMNI run/walk ten-point scale, ranging from no exertion to extreme exertion.^17^ Measures of thermal sensation, thermal comfort, and thermal preference are perceptual scales for assessing physical environments from the International Organization for Standardization (ISO; Geneva, Switzerland).^18^ These scales can be used to determine how participants feel about the thermal environment. Thermal sensation assesses what thermal environmental stimuli are impacting them at that moment on a nine-point scale (very cold to very hot), thermal comfort assesses how they feel about the impact of those stimuli on a five-point scale (comfortable to extremely uncomfortable), and thermal preference assesses what temperature they would prefer to be at in that moment on a seven-point scale (much cooler to much warmer).^17^

For both the cold (-8°C) and the neutral (20°C) conditions, the subjects performed an exercise treadmill protocol that simulated the aerobic demand on firefighters.^19^ At the start of each condition, baseline HR, RR, RPE, thermal comfort, thermal sensation, thermal preference, Tc, MST, and hand/finger temperature were recorded. These measures were re-assessed every five minutes for the duration of the trial and through the recovery interval. The subjects walked at 4.5kph on a 2.5% incline. After 20 minutes, the treadmill (Trackmaster; Newton, Kansas USA) was lowered to 2.5kph at a 0% incline for three minutes to mimic exiting the incident, followed by a four-minute standing period to simulate having a SCBA cylinder changed. Following the standing period, the subjects walked again at 2.5kph at a 0% incline for three minutes, followed by a 20-minute period of walking at 4.5kph at a 2.5% incline to simulate returning to the incident for a second work interval. The total protocol length was 50 minutes per condition, or when one of the termination criteria was met: (1) RR > 60 breaths/minute; (2) HR exceeding age-predicted maximum on two consecutive measurements; (3) Tc > 39.5°C or < 35°C; (4) unsteady gait making it unsafe to continue treadmill exercise; or (5) subject request.

At the end of each condition, subjects exited the chamber, doffed the TPC in a thermoneutral environment, immediately re-dressed in dry clothing, and were weighed. Subjects were then instructed to rest in the thermal neutral environment in a seated position for 20 minutes. They received a rehydration volume of room-temperature water (500mL) or electrolyte replacement (590mL - Gatorade, PepsiCo Inc.; Purchase, New York USA) based on participant preference. Vital signs, temperatures, and perceptual scales were recorded every five minutes for the duration of the 20-minute recovery interval.

### Statistical Analysis

Physiological measures and participants’ perceptual responses were analyzed for duration (time) and condition (thermal neutral/cold) during exertion and recovery. The MST was calculated using the formula: MST = chest (0.25) + back (0.25) + thigh (0.3) + arm (0.2).^15^ Statistical analyses were conducted using SAS software (version 9.4, SAS Institute Inc.; Cary, North Carolina USA). A Mixed-Effects Model for Repeated Measures (MMRM) was fit to compare the Tc between cold and neutral condition at time zero (T0; onset), time 20 (T20), and time 50 (T50; completion). The MMRM includes time, condition, and interaction term of time and condition. An unstructured covariance matrix was used to model the within-subject correlations across timepoints, with repeated measures specified for each subject-condition combination. Bonferroni correction was applied to adjust the confidence intervals (CI) and control the family-wise error rate for the three comparisons between conditions at each timepoint, using an adjusted significance level of 0.0167 (0.05/3).

## Results

Of the 20 subjects who began the study, six (30%) were lost to attrition: one (5%) for personal reasons, two (10%) for unrelated medical reasons, and three (15%) subjects requested to stop a protocol early or exceeded their age-adjusted HR maximum. These three subjects were removed for safety reasons and were not included in the analysis due to the lack of full data. Fourteen subjects (70%), including two (10%) females, completed the full study protocol and were included in the analysis. This analysis was not planned or powered for formal hypothesis testing. The observed data from 14 individuals were used to assess, while exercising in firefighting TPC, whether a cold environment helped maintain normal core body temperature and lowered extremity temperature compared to a thermally neutral environment. Mean demographics of the group were: age 30.6 years (median: 30.0, IQR: 9.0); mass 86.1kg (median: 83.5, IQR: 39.7); height 174cm (median: 176.0, IQR: 11); and BMI 28.1 (median: 27.2, IQR: 7.7). The temperature of the chamber was set to either 20.0°C or -8.0°C and was maintained at 20.0°C (median: 19.6, IQR: 0.6) for thermal neutral and -6.7°C (median: -6.9, IQR: 1.27) for cold conditions.

### Temperature Comparisons between Thermal Neutral and Cold

Core temperate under the cold condition was significantly lower than the thermal neutral condition at T50 (Diff: -0.30; Bonferroni-adjusted CI: [-0.5975, -0.00817]; P = .0142, with a mean difference of 0.3°C).

**Figure 2.**
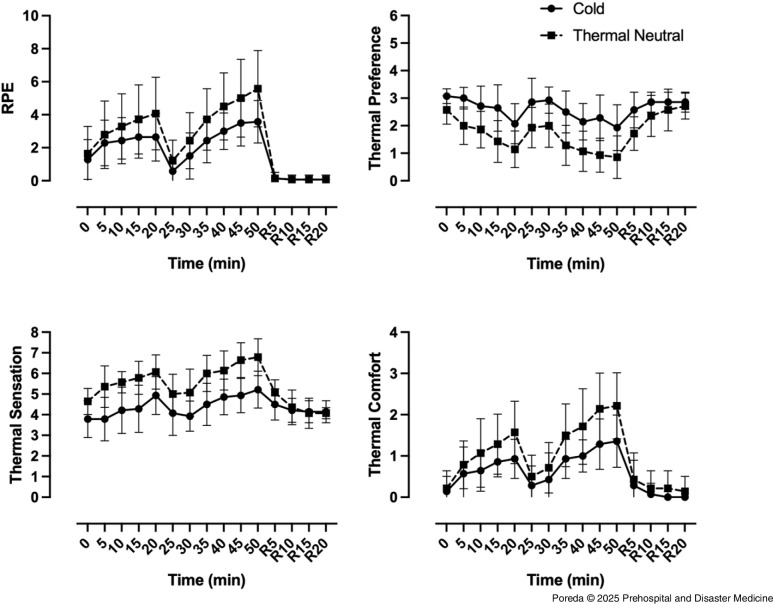
Heart Rate, Respiratory Rate, Core Temperature, and Mean Skin Temperature Responses to Treadmill Walking in Cold (Solid) and Thermal Neutral (Dashed) Conditions. Note: Data shown as mean (SD). Abbreviation: RPE, rate of perceived exertion.

The MST increased compared to T0 in the thermal neutral condition and T20 in the cold condition, but not to T50 in the cold condition. When comparing MST across conditions, T50 of the cold condition was lower than in the thermal neutral condition at T20 and T50 with mean differences of 2.8°C and 3.4°C, respectively (Figure 2). In the extremities, skin temperature increased from T0 to T50 during the thermal neutral condition on both hands and fingers. During the cold condition, finger temperature increased from baseline T0 to T50, but hand temperature did not change. The mean temperature of the hands was higher than the fingers for both conditions.

### Heart and Respiratory Rate Comparisons between Thermal Neutral and Cold

Cardiorespiratory responses to the workload and temperature were assessed. Increases were observed from onset (T0) to T20 and completion (T50) during each condition for both HR and RR, using T0 of neutral condition as the baseline reference (Figure 2).

Heart rate and RR were higher at the end of the thermal neutral condition when compared to the end of the cold condition, which indicated a greater physiological response to the temperature despite the workload remaining constant. The cold condition HR and RR at T50 were similar to the thermal neutral condition at T20.

### Perceptual Comparisons between Thermal Neutral and Cold

**Figure 3.**
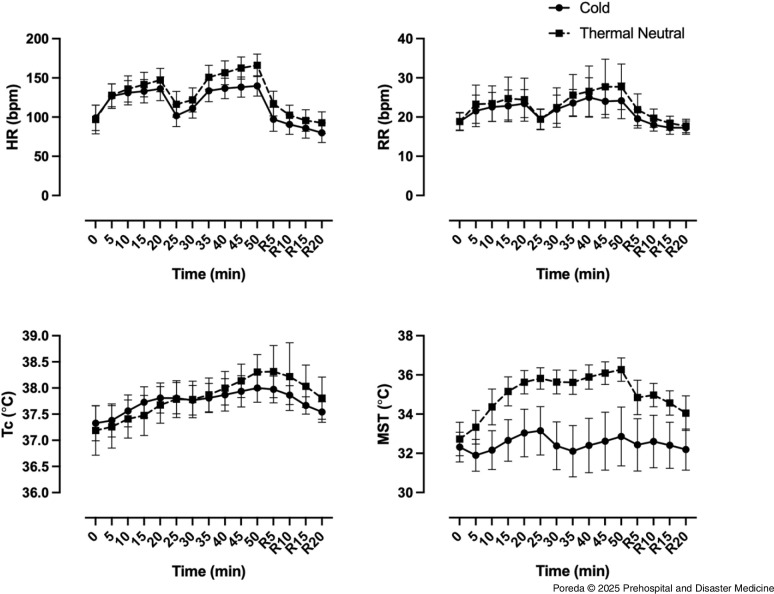
Perception of Effort (RPE), Temperature Preference, Thermal Sensation, and Thermal Comfort during Exertion in TPC in Cold (Solid) and Thermal Neutral (Dashed) Conditions. Note: Data shown as mean (SD). Abbreviations: TPC, thermal protective clothing; HR, heart rate; RR, respiratory rate; Tc, core temperature; MST, mean skin temperature.

During both conditions at T50, subjects perceived that they were working harder and experienced greater discomfort when compared to baseline T0. In addition, subjects reported both warmer thermal sensation and that they preferred to be cooler after 50 minutes of exertion, compared to baseline (Figure 3).

However, perceptual measures consistently indicated work in the cold condition while wearing TPC was easier and more comfortable than the thermal neutral condition. By the end (T50), the thermal neutral condition was higher for RPE, thermal comfort, and thermal sensation and lower for temperature preference when compared to the cold condition. Subjects rated 50 minutes of exertion in TPC in the cold condition similar to the 20-minute mark of the thermal neutral condition for RPE and comfort. Thermal sensation at the 20-minute mark of the thermal neutral condition was higher and temperature preference was lower than at the 50-minute mark of the cold condition, indicating they felt warmer and wanted to be cooler.

### Change in Body Mass

There was a significant mass loss during the cold condition, but the magnitude of change was small in both the cold (0.26kg) and the thermal neutral (0.53kg) conditions. These were not different between conditions and were substantially lower than that previously reported for higher intensity exertion in the heat.^19-21^

### Timing for Rehabilitation and Return to Work

Upon completion of the exercise protocol, all subjects completed a 20-minute recovery interval. Thermal sensation took longer to return to baseline in the cold condition. In both conditions, thermal comfort and thermal preference returned to based line by rest five-minute (R5) and rest ten-minute (R10), respectively (Figure 3). In both the cold and the thermal neutral conditions, Tc was not different from baseline at R10, which potentially indicated there was a mismatch between thermal perception and body temperature when exiting from a cold environment to a thermal neutral recovery area. The MST was not different from baseline during work or rehab in the cold condition, but took longer than 20 minutes to recover in the thermal neutral condition. Both HR and RR recovered sooner in the cold condition (Figure 2).

## Discussion

The current study hypothesized that wearing firefighting TPC in a cold environment would maintain normal core body temperature compared to a thermal neutral environment, and that extremity temperature would decrease in the cold environment. The results from this study proved more nuanced. Working in TPC did increase Tc in the thermal neutral environment, increasing slightly more (0.3°C) than in the cold environment Tc by the end of the work (T50). Extremity temperature in the cold environment initially decreased at the onset of the work (T0). However, as physical exertion caused an accumulation of body heat, the extremities re-warmed and most surpassed the initial (T0) temperatures by the end of the work conditions (T50).

### Physiological Responses to Exercises Under Thermal Neutral and Cold Conditions

In this moderate-intensity exertion, MST was lower in the cold condition when compared to the thermal neutral condition (3.4°C). The Tc was also lower, but to a lesser magnitude (0.3°C). The MST contributes to a person’s thermal comfort, while the physiological responses to temperature changes are more determined by Tc.^22^ The cold conditions used in this study have a moderate protective effect and slowed the rise in body temperature, but extrapolation of current data indicates sustained moderate-intensity operations would still result in hyperthermia and hypohydration. The more gradual rise in Tc in the cold suggests that firefighters may also be able to work longer in cold conditions without deleterious effect. It is worth noting that the conditions simulated working outdoors in TPC (ie, performing exterior firefighting tasks). It is likely that interior fire suppression would result in similar heat stress to other times of the year. The current study cannot explain how body temperature changes while wearing TPC during no/low-intensity work in the cold. Hand and finger temperature initially dropped in the cold condition before exertion caused them to rise above baseline (T0) temperatures, indicating that peripheral temperature of a gloved, dry hand can be preserved during exertion in these environmental conditions.

Rissanen, et al evaluated work in the cold while wearing chemical protective clothing.^11^ Their study showed that Tc was more related to activity level than environmental temperature, while finger temperatures were dependent on environmental temperature.^11^ Core temperature responses in the present report were similar to Rissanen, et al, but the extremity temperatures differed as they increased after heat accumulated during exertion in the current study.^11^ This is likely due to the type of gloves worn with TPC (heavy insulated leather) versus chemical protective ensembles (ie, butyl, nitrile); TPC gloves are specifically designed to resist temperature changes for structural firefighting, whereas chemical gloves are designed to resist chemical interactions for hazmat events.^3,23^

### Subjective Perception of Differences between Neutral and Cold Thermal Conditions

Subjects felt more comfortable in the cold environmental condition and preferred it over the thermal neutral environmental condition, despite the small difference in Tc. Similarly, thermal comfort and thermal sensation returned to baseline values before Tc. Together, these findings indicate that thermal perception is not a reliable indicator of body temperature, potentially leading firefighters to over-extend work or return to work prematurely.

### Emergency Incident Rehabilitation After Working in TPC in the Cold

The recovery process was faster and firefighters returned to their baseline HR and RR within five minutes for the cold condition compared to 10 minutes for the thermal neutral condition; this was related, at least in part, to lower peak HR and RR in the cold condition. This was also seen for MST, returning to baseline faster in the cold condition. These findings are expected for moderate-intensity exertion in TPC and differ from high-intensity exertion where recovery is typically incomplete even after 30 minutes.^20^

It is well-established that at hot temperatures, two work intervals are difficult and require prolonged recovery.^1,2,24,25^ The current study showed that 50 minutes of work in the cold was equal to or less strenuous than 20 minutes of work in the thermal neutral condition for most measures. While this study was not specifically designed for this analysis, this raises the question of how long firefighters can work safely at different temperatures, potentially allowing for reconsideration of manpower requirements and other fireground operational factors. An important consideration, however, is the misjudged thermal perception and the potential for over-exertion, which will require future research.

Key operational findings include that despite cold temperatures, hyperthermia and hypohydration remain significant concerns for firefighters, often unnoticed by the firefighters themselves, who may also suffer from digital frostbite. Core temperatures did not rise as much in cold conditions compared to thermal neutral temperatures, suggesting the potential for extended work periods and reduced manpower needs. Further studies will be required to substantiate this claim. The NFPA 1584 recommends additional warming layers or blankets to restore normal body temperature, which may hinder over-heated firefighters. Frostbite remains a risk alongside core hyperthermia. Further research into optimal rehabilitation methods is needed to address digital frostbite and core hyperthermia effectively. This study, while not focusing on rehabilitation methods, found that neutral temperature and gear removal allowed all subjects to return to baseline within 20 minutes.

## Limitations

A limitation of the current study is the reliance on laboratory-based exertion protocols, which do not fully replicate the variable physical demands encountered on the fireground. The temperature-controlled chamber also excludes external factors such as wind, water, and contact with cold tools or other materials, limiting the study’s real-world application. However, the advantage of higher resolution physiological data in the laboratory study partially offsets these limitations. Fluid loss measurements may have been influenced by not using nude weight. The impact was limited by having the subjects change into similar dry clothes for post-exertion weight measurement. Only healthy firefighters were selected for this study protocol to protect against any untoward consequences; however, this is not reflective of the general firefighter population who may have multiple medical problems that put them at risk of hyperthermia or frostbite. Secondary outcomes were analyzed descriptively. Six subjects failed to complete the study, including two subjects who exceeded their preset safety thresholds for HR. Excluding less physically fit individuals may further impact application of study results. These factors were incorporated to ensure the safety of the participants but reduce the generalizability across the fire service, where many firefighters are older and may have pre-existing medical conditions. This study did not evaluate fitness prior to the sessions and did not determine how long working activities might be extended, nor does it take into account the risk of frostbite due to prolonged exposure to the cold ambient air. Finally, this study has limited value to areas of the country that do not experience severe cold.

## Conclusion

Exertion in a cold environment while wearing TPC resulted in lower peak values and faster recovery for HR, RR, MST, and subjective measures when compared to the thermal neutral condition. These findings may allow for longer work intervals in cold environments prior to firefighters needing to go to emergency incident rehabilitation, or shorter rehabilitation times prior to returning to fireground operations. Leaders should also consider the risk of other cold-induced conditions due to prolonged exposure, such as frostbite, when developing rehabilitation protocols. Future research should investigate optimal recovery times, and the influence of wind, water, and contact with cold tools/objects to further assess the detrimental effects of operating in cold environments and being exposed to cold environments after interior fire suppression.
